# Biochemistry and physiology within the framework of the extended synthesis of evolutionary biology

**DOI:** 10.1186/s13062-016-0109-6

**Published:** 2016-02-09

**Authors:** Angelo Vianello, Sabina Passamonti

**Affiliations:** Dipartimento di Scienze Agrarie e Ambientali, Università degli Studi di Udine, 33100 Udine, Italy; Dipartimento di Scienze della Vita, Università degli Studi di Trieste, 34100 Trieste, Italy

**Keywords:** Living species, Evolution, Homeostasis, Molecular mechanisms

## Abstract

Functional biologists, like Claude Bernard, ask “How?”, meaning that they investigate the mechanisms underlying the emergence of biological functions (proximal causes), while evolutionary biologists, like Charles Darwin, asks “Why?”, meaning that they search the causes of adaptation, survival and evolution (remote causes). Are these divergent views on what is life? The epistemological role of functional biology (molecular biology, but also biochemistry, physiology, cell biology and so forth) appears essential, for its capacity to identify several mechanisms of natural selection of new characters, individuals and populations. Nevertheless, several issues remain unsolved, such as orphan metabolic activities, i.e., adaptive functions still missing the identification of the underlying genes and proteins, and orphan genes, i.e., genes that bear no signature of evolutionary history, yet provide an organism with improved adaptation to environmental changes. In the framework of the Extended Synthesis, we suggest that the adaptive roles of any known function/structure are reappraised in terms of their capacity to warrant constancy of the internal environment (homeostasis), a concept that encompasses both proximal and remote causes.

**Reviewers:** Dr. Neil Greenspan and Dr. Eugene Koonin.

## Background

In a paper that appeared more than 50 years ago, the renowned evolutionary biologist, Ernst Mayr, while discussing the term “biology”, realized that this field was perceived by the scientific community in a uniform and unified sense [1]. However, moving beyond a merely descriptive approach, very different fields of research emerged. For this reason, he suggested to organise biology into two major areas: *functional biology (physiology)* and *evolutionary biology.* Functional biology is concerned with the operation and interactions of structural components, starting from the molecular level, advancing to cells and organs, and eventually ending with individuals. The main question in this discipline is “how?”, or in other words, how does something function or operate? Functional biology analyses the proximal causes that we can experience every day in our lives. To do this, it utilizes several approaches, including disciplines such as biochemistry, physiology, genetics, cell biology and so forth. Evolutionary biology faces different problems and, hence, adopts methods other than those of functional biology. In this case, the basic question refers to what we call remote (or finalistic) causes. However, when we ask “why?” we must be aware of the ambiguity of the questions that lie behind “deep” (geological) time. In fact, can we provide answers concerning early life by just incrementally increasing our knowledge about current conditions? The reason for these divergent questions might be the lack of interaction between Charles R. Darwin (the founder of evolutionary biology) and Claude Bernard (the founder of modern physiology), albeit both lived during the same time period.

This gap between functional and evolutionary biology is however, closing, thanks to the expansion of the *repertoire* of molecular mechanisms, which, through a better knowledge of the chemistry and biology of nucleic acids, provides mechanistic details (the “how”) supporting the plausibility of the “why”, i.e., the remote causes. Therefore, evolutionary biologists are now able to describe evolution from both perspectives, which are indeed complementary. However, dark zones remain, such as missing links between the so-called *orphan metabolic activities* [2–4] and their as yet undiscovered, corresponding genes. Do orphan metabolic activities, amounting to more than 20 % of all known metabolic reactions, arise from unrecognized exaptations? Similarly, what functions do the so-called *orphan genes* play in the organisms [5]? The latter are genes that lack coding-sequence similarity outside their species. These questions are surrounded by epistemological frustration, yet they represent areas for true discovery. Thus, the interaction between functional and evolutionary biology must be strengthened.

In this review, we try to describe, from a historical point of view, the relationship between these two branches of biology. In particular, the increasing role of functional biology (genetics, biochemistry, molecular biology, physiology, etc.) in explaining evolutionary mechanisms in the framework of a new vision, called Extended Synthesis, will be described.

## Review

### From Darwinism to Modern Synthesis

Although *The Origin of Species*, by C.R. Darwin, was published over 100 years ago, natural selection still represents the most fundamental mechanism to explain the evolution of life [6]. The *Darwinian Theory,* also named *Darwinism*, is based on two pillars: i) a hereditable phenotypic variation exists within a population (species) on which ii) natural selection, acting on individuals, determines the sorting of the different members of the population. This theory has two major implications: i) living organisms are represented as being linked together in the *tree of life*, whose primary origin is grounded in an early organism, now called *LUCA* (an acronym for *Last Universal Common Ancestor*) [7]. According to Darwin, the process of species formation is gradual and progressive due to the cumulative addition of small traits.

At the beginning of the 20^th^ century, this view was challenged by knowledge emerging from genetics, a new discipline born after the rediscovery of the work of G. Mendel by three biologists, H. de Vries, E.C. Correns, and E. von Tschermak-Seysenegg. The main question, among others, concerned the evident discontinuity by which single genes are transferred and expressed from one generation to another, which is in conflict with the continuous trend of evolution, as hypothesized by Darwin. This challenge was confronted and resolved during the first part of the 20^th^ century by *Neo-Darwinism* or *Modern Synthesis* [8–12]. This new theory, which is the result of the contribution of several scientists, can be subdivided into two phases. The first phase, named Fisherian, which occurred during the initial decades, was mainly the result of the efforts of a group of mathematician-geneticists (E. Fisher, J. B. S. Haldane and S. Wright). The main postulate was the explanation of evolution in terms of changes in frequencies of the alternative forms of genes (alleles) in a population. In this way, natural selection together with hereditary variation could become the only determinant in evolution. However, this satisfactorily explains how a population (species) adapts to its environment (*adaptationism*), but does not explain how a new species arises from a pre-existing one. Several naturalists, including E. Mayr, T. Dobzhansky, J. Huxley, J. G. Simpson, and J. L. Stebbins addressed this issue only in the second phase. To answer to this crucial question, E. Mayr and T. Dobzhansky introduced the concept of *allopatric speciation*. According to this concept, a new species arises when a daughter population separates from the mother population by different causes (geographic, genetic, etc.), such that the individuals of the two populations are no longer able to interbreed. As a result, both populations undertake divergent evolutionary trajectories, leading to the development of two different species.

In the middle of the 20^th^ century, the *Modern Synthesis Theory* seemed to have clarified all the questions raised by evolutionary biology. Thus, every discussion on the mechanisms of evolutions was hardened. Indeed, still in 1977, François Jacob asserted that natural selection is the result of two constraints: i) the requirement of genetic variability, which is generated by specific genetic mechanisms (i.e., mutation, recombination) and sexual reproduction to generate similar, but not identical individuals, and ii) the requirement of a constant exchange of matter, energy and information between the organism and the environment [13].

### Towards an extended (pluralistic) synthesis

The *Modern Synthesis Theory* thus became a paradigm in evolutionary biology. This perspective, in line with the Darwinian theory, still implies that evolution is a continuous and linear process, because a new species would arise by cumulative addition of several, but small changes (favorable mutations).

However, this scenario changed radically in 1971, when S. J. Gould and N. Eldredge introduced the *Punctuated Equilibria Theory* [14]. According to this view, evolution may be described as a phenomenon featured by long periods of stasis in which species are stable, interrupted (or “punctuated”) by short periods characterized by rapid changes leading to the formation of new species. The major conceptual change provided by the *Punctuated Equilibria Theory* is related to the recognition of the role played by *contingency* in evolution alongside the element of *chance*. While contingency arises from the interplay of several environmental factors, chance (i.e., a mutation) is related to a single “all or nothing” phenomenon, like throwing dice. The discovery that today’s living organisms have survived not less than five extinctions after the Cambrian period is strongly in favor of a role for contingency and discontinuity against continuity and gradualism in evolution [15].

A good example of contingency is offered by *endosymbiosis*, whose role in the evolution of life was perceived between the end of the 19^th^ and beginning of the 20^th^ century, when a group of scientists (A. F. W. Schimper, A. Meyer and C. Mereschkowsky) postulated that plastids, organelles found in eukaryotic cells, derived from the inclusion of certain bacteria into larger eukaryotic hosts. However, a complete theory on the origin of mitochondria and plastids by endosymbiosis was launched only at the end of sixties of the last century [16]. Nowadays, it is accepted that both mitochondria and plastids arose by a single endosymbiotic event involving, respectively, an alpha-proteobacterium and a cyanobacterium [17]. In particular, plastids evolved in a subset of eukaryotes by so-called *primary* endosymbiosis, with an ancient cyanobacterial lineage. Subsequently, this primary endosymbiont spread to other eukaryotes by *secondary* or *tertiary endosymbioses* [18].

In 1982, S.J. Gould and E.S. Vrba reintroduced a missing term in evolutionary biology [19]. Starting from pre-adaptationism, a principle already conceived by Darwin, they proposed a distinction between *adaptation* and *exaptation*. Adaptation, as known, identifies a character for a current use, which is shaped by natural selection. Exaptation may be obtained in two ways. First, a trait is shaped by natural selection to perform a particular function (an adaptation), and then it is co-opted for a new use. A classic example of this type of exaptation is offered by the feathers of birds, which initially evolved as a way to ensure thermoregulation, but were later co-opted for flying. Alternatively, a character whose origin is not related to natural selection (nonaption) is co-opted to accomplish a function for a current use.

The concept of *niche construction* was launched by R. Lewontin [20] and rapidly became a relevant field of research in evolutionary biology [21]. Examples of this phenomenon are widespread, ranging from birds that build their nest to beavers building their dams, until the thermoregulation of termitary and their nests performed by social insects. At a different level, bone marrow provides distinct niche environments in which phenotypically identical cells display different functions according to the area of the bone marrow in which they reside [22]. But it is in human populations in which niche construction displayed a major function. This underlines the role of culture in evolution – intended as a means to modify the environment in which a population lives. Although R. Dawkins interprets this mechanism in a reductionist fashion, named extended phenotype [23], niche construction, particularly in the case of humans, represents a major device in shaping evolution [24].

*Cooperation* is also needed during evolution to build up new levels of complex organizations, ranging from genomes, cells, multicellular organisms, social insects and human society. Cooperation, often improperly named altruism, can result, according to M.A. Nowak, via five mechanisms: kin selection, direct reciprocity, indirect reciprocity, network reciprocity and group selection [25]. Cooperation would arise by repression of competition within groups, thus enhancing group success in competition against other groups. This interpretation has been suggested in the framework of kin selection and would be involved at different levels for the vast majority of living organisms, such as meiosis, metazoan success and human sociality [26].

Historically, epigenetics faces aspects that E. Mayr named “soft inheritance”. In other words, these are new variations induced by the environment that are transmitted to the next generations. One of the most relevant conquests of the last few years is represented by the rediscovery of epigenetics in the framework of molecular biology. Epigenetics can be now defined as “the structural adaptation of chromosomal regions so as to register, signal or perpetuate altered activity states” [27]. This type of modification may involve DNA methylation, while histones may be either methylated or acetylated. In a more extended view, it is useful to distinguish epigenetics from epigenetic inheritance. The first term is concerned, as above seen, with regulatory mechanisms leading to inducible and persistent changes occurring during development. The second term refers to both body-to-body (soma-to-soma) information transfer, arising from mother and offspring interactions, and cellular epigenetic inheritance. It consists in transmission of variations from mother cells to daughter cells, beyond DNA differences or persistent inducible signals in the environment of the cell. This mechanism is widespread, occurring in mitotically dividing cells of prokaryotes and protists, but also in pluricellular eukaryotes, as well as during meiotic cell divisions in germ-lines to produce sperm or eggs. In this context, Jablonka and Raz [28] distinguish four types of *Epigenetic Inheritance Systems* (*EIS*), an acronym suggested by J. Maynard Smith. These systems include mechanisms for: (1) self-sustaining regulation loops; (2) structural templating; (3) chromatin marking; (4) RNA-mediated inheritance. Hence, *EIS* provide rapid devices to increase variations within a population, superposed on the well known genetic variations, thus increasing the whole variability of a population on which natural selection acts.

The importance of epigenetics in shaping evolution is still under debate. However, there is increasing evidence suggesting that epigenetic changes may contribute to the evolution of species [28]. Not only that. Epigenetic variations may be transmitted by both germ-line and soma-to-soma mechanisms. These changes, being conserved from a generation to another, may contribute to evolution [29, 30]. Thus, natural selection could then choose the most adaptive variations arisen in the genome, the epigenome and so forth [31]. In such a way, epigenetics rescues some aspects of the Lamarkian theory.

### Is the number of evolvable species finite or infinite?

It is not possible to affirm that natural selection can induce the origin of any type of organism, because matter has several *structural constraints.* This aspect is crucial, being at the basis of the origin and development of forms [32]. The main question concerns the capacity to evaluate the probability distributions of alternative phenotypes towards which an organism could evolve. In this sense, the first significant effort has been made by *evo-devo* (an acronym for *evolutionary developmental biology*). This new branch of biology arose during the nineteenth years of the last century. This revolution was anticipated by S. J. Gould in his milestone book, *Ontogeny and Phylogeny*, published in 1977 [33], but was experimentally validated later by the contribution of several scientists [34–37]. Albeit not necessarily satisfactorily, evo-devo is often described as the genetic and physiological processes – in particular, regulatory genes involved in development – occurring during the development of an organism. This definition has to be expanded by focusing on the evolution of development and, in particular, on evolutionary innovations and evolvability, intended as the probability of a trait to change, in evolution, into any, or all, of the alternative imaginable phenotypes [38]. As clearly stated by A. Minelli, evo-devo is not extraneous to the neo-Darwinian perspective, because development can be profitably integrated within this evolutionary mechanism. However, most importantly, evo-devo is not simply developmental biology grafted onto evolutionary biology, but rather it is a new field of research with a specific conceptual endowment and program [38]. Indeed, what we can learn from evo-devo goes beyond these statements. Every trait of a species possesses a probability to change into any, or all, of the alternative phenotypes of the evolutionary landscape (*evolvability*). Moreover, the rules of the game may change through time. In light of evo-devo, evolution could be conceived as a change in ontogenetic processes, rather than genotypic or phenotypic changes.

These new interpretations of the evolutionary mechanisms have triggered heated debates that are still ongoing [39]. On the one hand, we can find neo-Darwinists, who, with different degrees of emphasis, contend that evolution may be explained only within the framework of the modern synthesis [40–42]. On the other hand, the pluralistic attitude of naturalists, though not refusing Darwinism and Modern Synthesis, affirms that Neo-Darwinism must be integrated using the most recent pieces of knowledge in a new paradigm, termed the Extended Synthesis [39, 43, 44]. This debate is ongoing, as researchers are still divided on the necessity to either rethink or drop the evolutionary theory [45].

### Functional biology and modern synthesis

The gap between Mayr’s two branches of biology was perceived throughout the 20^th^ century. Functional biology studies rarely consider the functions examined in the framework of an evolutionary context, which is more often confronted by genetic approaches and also currently by molecular biology. But even in the latter cases, the reasoning is developed almost exclusively within a strict neo-Darwinian perspective; that is, only in the light of natural selection. Functional biology studies, in particular by employing biochemistry and molecular biology approaches, show that Modern Synthesis serves as a useful framework in which to obtain evolutionary explanations of the functions studied, though, most often, the contents of this theory are not adequately examined and discussed [46]. Studies are strongly influenced by reductionism, which limits the capacity to perceive the phenomena in a holistic context. Nevertheless, molecular biology has provided a great contribution towards understanding the basic physico-chemical mechanisms operating in evolution.

The contribution of functional biology – in particular molecular biology – to evolutionary biology, can be subdivided into two branches: i) new evolutionary models [46], and ii) new sources of genetic change. The first fundamental mechanism is represented by *gene duplication*, which is followed by mutations, leading to the divergence and functional specialization of the two copies. Duplications are not limited to the gene level, but can occur also at chromosome and genome levels [47]. A completely different mechanism involves *loss of function*. Initially identified by W. Bateson [48], this modality was strongly supported by A. Lwoff, who demonstrated that evolution of living organisms is also linked to the loss of some metabolic activities and their corresponding genes [49]. Although verified in different cases, this mechanism generates a paradox, because it implies that the first organism would have had many metabolic functions linked to unknown (and unknowledgeable) genes. Thus, those experiments aimed at synthesizing the minimal cell should be seen as technological performances, rather than attempts at understanding the origin of life. Notwithstanding this lack of evidence, it can be speculated that the divergence between *Bacteria* and *Archaea* from LUCA would have been achieved by the selective loss of functions that were initially present in LUCA [50]. Further mechanisms include *addition* and *complexification*, well exemplified by the formation of metabolic cycles and, in particular, the Krebs cycle. As suggested by J.B. Baldwin and H. Krebs, this cycle could be constructed by the assembly of pre-existing linear pathways [51]. The well-known *recombination of DNA fragments* (exons) is another mechanism that generates different sequences of mRNA (alternative spicing), leading to several proteins from the same single gene [52]. The last mechanism probably plays the most important role in evolution in terms of originating new variability, as it involves *mutations of regulatory genes* whose products modulate the expression of structural genes [53].

Both the Darwinian Theory and the Modern Synthesis assume that all phenotypic variability depends on genetic mutations. As a consequence, mutations are the pre-requisite for natural selection to occur. This scenario was amplified and modified in the second part of the 20^th^ century by the discovery of several mechanisms capable of challenging or extending this view. One of the major accomplishments was the sequencing of the human genome [54, 55]. However, human beings were found to have only slightly more than 20,000 genes, which was unexpectedly less (rather than more) than several animals [56], with the number of nucleotides in the genes accounting for approximately 2 % of the total number of nucleotides in the entire genome. The remaining part of the genome was simply identified as “junk” or “waste” DNA. Fortunately, as described above, one gene can code for several protein variations via the mechanism of alternative splicing. A part of “junk DNA”, on the basis of experimental and biocomputational efforts, was found to be responsible for the synthesis of a plethora of untranslated (non-coding) RNAs, including miRNA, snoRNA, and siRNA species [57]. However, most importantly, these molecules appear to possess crucial regulatory roles in cells, even during developmental stages [58]. Moreover, “superfluous” DNA, aberrant transcripts or processing products bear a vast evolutionary potential through sorting via natural selection.

Another mechanism generating genetic variability is the insertion of RNA modules into DNA by *retrotransposition* [59]. This modality gives support to an early hypothesis that this mechanism could represent an “echo of the past”, recognized as a ribonucleoprotein (RNP) world [58]. In this new scenario, the function of transposones, or jumping genes, was reconsidered, because these transposones can represent a further source of genetic variability capable of playing a role in evolution [60]. In an interesting paper on horizontal gene transfer, L. Boto concludes that “there is a need for a new evolutionary paradigm that includes horizontal gene transfer and other mechanisms in the explanation of evolution” [61].

We can conclude that, besides mutations, cells have a number of mechanisms to keep genomes in flux and thus to generate inter- and intra-specific variations that are useful enough to be selected during evolution. Furthermore, there are several molecular modes by which evolution can be shaped. This new knowledge, however, does not substantially contradict modern synthesis, but rather helps explaining the dynamics of evolution. Nevertheless, several questions remain.

## Discussion

### Functional biology in the framework of the extended (pluralistic) synthesis

One of the major challenges to modern synthesis is perhaps the *neutralist theory* proposed by M. Kimura, who showed that the majority of gene substitutions occurring in organisms is the result of a random fixation of neutral or nearly neutral mutations; hence, they are without an apparent (immediate) selective value [62].

The discovery of pseudogenes represents another aspect, which can help us to better understand evolution in an extended view. Pseudogenes may arise solely by DNA duplication or RNA retrotransposition, without the subsequent influence of natural selection [63]. In the first case (duplication), pseudogenes are composed of exons and introns, whereas in the second case (retrotransposition), they contain only exons. Pseudogenes were considered non-functional (fossil) genomic sequences, but now evidence suggests that many of them might perform some type of biological activity [64]. The regulatory role of pseudogenes on transcription expands the perspectives to better understand the molecular bases underlying large phenotypical differences shown by species whose genes are very similar [65]. This recovered activity is promoted by mutations that incorporate new functions, which are then subjected to natural selection. In the conceptual framework of Modern Synthesis, molecular biologists are able to better describe micro-evolution (origin of species), but not always macro-evolution (origin of clades). In line with this, molecular biologists inadequately explored the evolvability of living organisms (range of possible phenotypes). A major part of these phenotypes are apparently forbidden, even in the absence of arguments for their adaptive value [37]. On the contrary, others (“monstrous”), without an evolutionary future, are constantly generated by point mutations. The latter observation was still made by R. Goldschmidt several decades ago [66]. Although initially refuted, these ideas were eventually rejuvenated [19, 67], leading to a hypothesis supporting a fundamental role of regulatory genes in evolution, a crucial catalyst for the birth of evo-devo, as described above.

Another major challenge arose, paradoxically, after the genomic revolution, with the discovery of *orphan metabolic activities* and *orphan genes.* A significant fraction of known metabolic activities, though well characterized phenotypically, are termed “orphan”, because they do not correspond to any known gene(s). Initially, they were estimated to be as many as 38 % of all [3, 4], a figure recently updated to 22 % (26 % in Eukaryota) [2, 68]. The inability to understand the genetics underlying a given phenotype (i.e., the orphan enzyme activity) is a major limit to understanding the fine mechanisms of evolution. This has tangible implications, since those same mechanisms may determine the success of new biotechnological products [69], or of new therapeutic approaches to win the global challenge of cancer [70].

Orphan genes are so named because they are present in one lineage, but do not possess homologues elsewhere.

Knowledge on their evolutionary origin is still scarce, but they possibly arose by duplication and rearrangement followed by divergence [5], *de novo* origin; e.g., from noncoding genomic regions [71], exaptation form transposable elements, and other mechanisms [72]. Every evolutionary lineage contains orphan genes and, at the moment, their presence is interpreted as a source of raw material available for the rapid adaptation to environmental changes and the process of speciation. Though the turnover of orphan genes is high (i.e., the equilibrium of new genes formed and their inactivation by mutations leading to loss of transcription), their role in shaping the phenotype may be deduced by the fact that they represent as much as 30 % of known genes [73–75].

To this point, we have to recognize that the relationship between functional biology and evolutionary biology can be strengthened, because, as stated by T. Dobhzansky, “Nothing makes sense in biology, but in the light of evolution”. Thus, three fundamental assumptions might help improve understanding the origin and evolution of living organisms. First, as stated by E.S. Vrba and S.J. Gould, in a hierarchical world, different evolutionary entities/individuals (genes, organisms, species), at ascending levels of complexity, may be recognized [76]. Second, this hierarchical perspective offers the possibility to read and better interpret evolution as the product of tinkering, according to the suggestive metaphor of F. Jacob [13], which is particularly useful at the molecular and biochemical level. Third, by adopting the pluralistic view of the Extended Synthesis, a richer heuristic conceptual framework becomes available to interpret biochemical results in an evolutionary perspective.

Consistently, there are already some valuable examples concerning exaptation and cooperation. Exaptation has been largely recognized at the molecular level (i.e., lens crystallins) [77] and, recently, has been used to explain the origin of a mitochondrial function, called the Permeability Transition Pore, PTP [78], which is involved in programmed cell death and possibly in calcium homeostasis [79]. This could well be the start of a true exaptation program in biochemistry [80]. The value of exaptation in explaining biochemical innovations has been further confirmed by studying metabolism, an ancient system that is at the core of life [81]. A metabolic network for synthesizing all biomass from a single source of carbon and energy has been analyzed by a computational approach. When such networks are viable on a particular carbon source, they are viable on multiple other carbon sources that were not targets of selection. In other words, metabolic systems have a potential for non-adaptive innovations (exaptations) that surely shaped the evolution of life. Cooperation has also been largely recognized at the biochemical level (i.e., allosteric enzymes), but there are some recent interesting examples that are worth mentioning. The first example is the cooperation/competition of ATP-producing pathways [82]. Using an analysis of model situations and biochemical observations, these authors showed that a kind of metabolism that generates ATP production at a low rate and at high yield may be considered as a form of cooperative use of the resource, which may evolve in spatially structured environments (like a cell). This mechanism could have facilitated the transition from unicellular to multicellular organisms. Cooperation could also be at the basis of the universal use in living organisms of “left-handed” amino acids and “right-handed” sugars [81]. Evidence is now emerging on how these choices were fixed during evolution and how this specialization can be interpreted as a form to facilitate cooperation (rather than competition) among all species, so that the latter can activate free exchange of those kinds of molecules.

In this new context of the extended synthesis, characterized by a holistic approach, it is possible to contribute to eliminating the gap between functional biology (physiology) and evolutionary biology. To do this, it is crucial to abandon woeful reductionism, because, as stated by G.E. Billmann (who cited D. Noble), “a sequence of base pairs in the DNA molecule can no more explain the complexities of life than a series of 1 s and 0 s on a compact disc recording can explain the emotional response to music” [83].

The unifying concept could be what C. Bernard named “the constancy of the internal environment”. This idea was then re-taken by the American physiologist, Walter Bradford Cannon (1871–1945), who developed and popularized the concept of *homeostasis* [84], described as the complex interplay of signals and sensors by which the internal environment of an organism is kept constant, while the external conditions change. Therefore, the adaptation of a species to its environment could be better assessed in terms of homeostasis, rather than in terms of the single components of its body (genes, proteins, cells, organs). Its function mainly consists in minimizing physiological fluctuations that alter the transfer of information through physiological systems [85]. In such a way homeostasis could have a role in both ecology and evolution [86]. As suggested by C.G. Gross, there are the conditions for a *renaissance* of homeostasis in the framework of the (extended) evolutionary biology, because when “the gap between evolutionary thought and general physiology began to be closed through the comparison of constituents of sea water and bodily fluids at different phylogenetic stages, the constancy of the internal environment suddenly took on new and accessible meaning” [87]. In other words, Bernard meets Darwin.

## Conclusions

The extended synthesis of evolution theory offers a multidisciplinary framework in which several branches of biology are fully integrated to obtain an improved description of the evolution of life. In this view, functional biology appears to play an essential role. Genetics and molecular biology offer powerful tools to understanding the mechanisms underlying changes in the structures and functions observed in cells and organisms. Biochemistry and physiology, by measuring either single or multiple functions and assessing the structural elements of phenotypes, have also the ability to focus on different levels of the organization of living species. Besides that, biochemical methods enable to characterize both the biotic and abiotic factors, triggering homeostatic responses in living entities, thus leaping from a reductionist to a holistic perspective.

The concept of homeostasis could, therefore, drive various methods and disciplines towards closing the gap between functional and evolutionary biology.

## Reviewers’ comments

### Reviewer’s report 1: Dr. Eugene Koonin, National Center for Biotechnology Information (NCBI), National Library of Medicine (NLM), National Institutes of Health (NIH). Bethesda, MD 20894, USA

In this article, Vianello and Passamonti discuss the importance of integrating functional and evolutionary biology within the framework of an "Extended Evolutionary Synthesis". It is difficult to object to the major emphasis of the article (as I understood it), i.e., that biochemical constraints are important for organismal evolution but have not been actually taken into account in the Modern Synthesis, at least not explicitly. However, the authors do not truly assess the role of homeostasis in evolution and present very few specific examples. As such, the article is more of an essay than a review. The subject is quite complex and difficult, and Vianello and Passamonti offer an interesting, lucid discussion, so the publication of this article can only be welcomed. However, it is best to keep in mind that this is not even a sketch of the Extended Synthesis, only a discussion of some of its salient aspects.

The subject is vast and highly complex, so one could have any number of suggestions or a minimal number of immediately obvious ones. I am choosing the second option. My suggestions are all rather minor and pertain to the presentation.

1. I wonder whether it would be more appropriate and a better reflection of the actual content of the article to expand the title to "Biochemistry and Physiology within the Framework …" (yes, I think 'framework' here is better than 'frame').
2. The abstract is too brief and insufficiently clear. The 'orphan metabolic activities and orphan genes, forming a stock set at the margins of scientific knowledge' are introduced quite abruptly such that this part of the abstract, which is supposed to present the specifics of the authors' ideas, could puzzle the reader. It is necessary to explain and expand.
3. I do not fully agree with the dichotomy of 'how' and 'why' questions between functional biology and evolutionary biology. This view misrepresents evolutionary biology and even might hint at teleology, which is highly undesirable. Evolutionary biologists certainly care about the way evolution proceeds (a 'how' question) not only 'why' it happens. Thus, it is important to be more accurate in the description of the two complementary perspectives on the world of biology.
4. A minor point but I think it is not quite fair and could be considered inflammatory to claim that the Modern Synthesis has turned into a dogma. It might have "hardened", according to Gould, but not quite to the dogmatic state.

Authors’ response: *Thank you very much for the general comment. In particular, we have considered all the points raised and answered as follows:*

*General*

*We agree that the subject covered in our manuscript is vast and complex. That is the reason why we have preferred not to expand it by adding more examples on the role of homeostasis in evolution. Our aim was not just focusing on that topic; instead, it was evoking the physiological principle of homeostasis, which could bridge functional and evolutionary biology. However, we have better explained why the physiological principle of homeostasis may improve understanding evolution, as suggested by Reviewer 2 (see below).*

*Specific*

1. *The title has been amended.*
2. *The abstract has been completely rewritten with a better specification of the orphan metabolic activities.*
3. *The dichotomy of “how” and “why” has been removed both in the abstract and in the main text and replaced by proximal (functional) and remote (evolutionary) causes (see pages 3 and 4).*
4. *The term “dogma” has been exchanged with “hardened” (page 6).*

**Reviewer’s comment.** Comments have been addressed.

### Reviewer’s report 2: Dr. Neil Greenspan, Case Western Reserve University, Cleveland, OH 44106–7288, USA

I am not able to identify a good reason for publishing this review article, as I can find no novel insights, arguments, or useful proposals for further research.

General: Vianello and Passamonti have produced a review that argues for the importance of “functional biology” to a full appreciation of the evolutionary origins of any given life form. I was not under the impression that there was widespread doubt about the relevance of functional biology to evolutionary questions in the current research community. In their abstract, the authors refer to unresolved questions pertaining to orphan metabolic activities and orphan genes, but they actually address these issues exceptionally briefly only on pages 4 and 14. The authors do not offer answers to the challenges that they claim are posed to the understanding of evolution by orphan metabolic activities and orphan genes. Furthermore, little illumination is shed on the overall issues pertaining to orphan metabolic activities and genes and no experimentally useful guidance is offered for how investigators should or might further illuminate them. The preceding comments highlight a recurring feature of this manuscript: articles, concepts, and names of important individuals are cited with inadequate elaboration or connection to other similarly cited articles, concepts, and names of important individuals. Consequently, the authors do not obviously advance current understanding of biological evolution, and I did not gain much in the way of fresh insights from reading the manuscript. In addition, a number of questionable assertions are made (see below).

Specific: p. 5 – The authors claim that, “According to Darwin, the process of species formation is linear …” I can only speculate that by “linear” the authors mean that the mutations leading to speciation occur at a roughly constant rate, presumably their interpretation of

Darwin’s suggestion that evolution is “gradual.” I do not necessarily assume that by “gradual” Darwin assumed that the occurrence of mutations or those mutations relevant to speciation occur at an absolutely constant rate on all time scales. In any case, as is well known, Darwin did not have any insight into genetics so I see no point in making a great deal out of his expectation of gradualism (as many others pointlessly continue to do). p. 9 – The authors imply that epigenetic modifications are either methylations of DNA or acetylation of histones. In fact, histone modifications affecting transcription include both methylation and acetylation. p. 9 The authors suggest that environmentally induced epigenetic modifications are “conserved from one generation to another, so that they may contribute to evolution.” This statement fails to note the essential distinction between epigenetic modifications of genomes in somatic cells and those affecting genomes in germ cells. Only the latter are likely to have substantial influence on organismal evolution. The authors also fail to make clear that it may not be the case that all epigenetic modifications are conserved in progeny or that even some that are may be conserved only through a few generations and not an indefinite number of future generations. p. 12 – The authors indicate that “junk DNA” has now been shown to be the source of many untranslated RNA species that fall into different categories, such as miRNA and snoRNA. They should however note that not all DNA previously referred to as “junk” is the source of untranslated RNA with identified or identifiable functions. p. 14 – The authors assert that pseudogenes represent a challenge to the Darwinian view of evolution. Precisely what this challenge would be is left unspecified, and I do not immediately see why pseudogenes would be difficult to incorporate into the current neoDarwinian view of evolution. p. 14 – The authors state that within the framework of the modern synthesis, molecular biologists can investigate (“study”) the origins of species but not the origins of higher taxa (clades). Insufficient support or elaboration on the basis for this dubious claim, which turns on an unspecified definition for “study,” is forthcoming. p. 17 – The suggestion that adaptations are better interpreted with reference to homeostasis as opposed to particular genes, proteins, cells or organs, sets up a false dichotomy.

There is no issue with understanding adaptations with respect to both overall physiological mechanisms or imperatives and particular structural elements of the organism under consideration.

Authors’ response: *Albeit we cannot share his general comment, we found his specific questions very useful. Thanks, therefore. We tried to answer as follows:*

*General*

*The aspects related to both orphan genes and orphan metabolic activities have been extended, discussing the more recent literature (with 10 new citations) (pages 16–17). Therefore, the abstract has also been rewritten, as suggested by Reviewer 3.*

*Specific*

1. *P.5: The word “gradual” has replaced “linear” (page 5).*
2. *P.9: The paragraph dealing with epigenetics has been rewritten, also addressing the question of post-translational modifications of histones (page 9).*
3. *P.12: We have specified that “junk” DNA is not simply junk, because part of it is transcribed (page 14).*
4. *P.14: The pseudogenes are now considered as an aspect that may be interpreted in the light of neutral mutations. Thus, this is not a challenge to Neo-darwinism (page 15).*
5. *P.14 (bis): In the framework of the Extended Synthesis, the origin of new clades is better explained: consider the contribution of Evo-Devo.*
6. *P.17: Supported by two new references to the literature, we have better explained why the physiological principle of homeostasis may improve understanding evolution and also ecology (page 19).*

**Reviewer’s comment 1.** General: My major concerns pertaining to the original manuscript by Vianello and Passamonti remain incompletely addressed in this revised version. By coincidence, I have been reading and am about 85 % of the way through the book, “Evolution – the Extended Synthesis,” edited by Pigliucci and Müller (MIT Press, 2010). Consequently, I have had a recent and thorough exposure to the thinking of those who argue for amending the conventional perspective on evolutionary theory represented by the Modern Synthesis. I continue to find that this review fails to substantially advance the thinking in any of the numerous topics addressed by this volume.

Authors’ response: *The scope of this review is to suggest that biochemical and physiological data could be better interpreted in the framework of a “pluralistic” approach to evolution, such as that described in the book “Evolution – the Extended Synthesis”, which we have mentioned in the text (see reference* [44]*). Therefore, this review does not have the aim to “advance the thinking in any of the numerous topic addressed in this volume”. The intent is to attract the interest of researchers in physiology, biochemistry, molecular biology, and other sister disciplines characterized by a reductionist approach to describe phenomena of life. We feel that awareness of the various issues of evolutionary biology might add value to their findings, by enlarging their readership, or opening unthought-of perspectives.*

**Reviewer’s comment 2.** Calling attention to the issue of orphan metabolic activities and orphan genes is worthy, but this article does not sufficiently develop this particular focus so as to yield truly new theoretical insights, offer novel explanations for unexplained phenomena, or offer concrete proposals for new research. An article that touches only superficially on numerous aspects of the proposed extended synthesis is not needed given the aforementioned book and numerous other articles and books from the past 5–10 years. The authors could provide a service by focusing solely on orphan metabolic activities or genes and describing novel insights or proposing new experiments to address outstanding questions.

Authors’ response: *We are glad that the reviewer has appreciated our original intent to introduce the topics of orphan metabolic activities and orphan genes in the framework of the Extended Synthesis. There are already excellent reviews on orphan metabolic activities or genes, focusing on the scientific and technological challenges to characterize them either as known or as new general biological mechanisms.*

**Reviewer’s comment 3.** Illustrative Specific Issues: p. 4. In his 1961 article in Science, Mayr did not suggest organizing “biology into two major areas.” His assessment that there are two sorts of biology, functional and evolutionary, was descriptive not prescriptive. Of course, Mayr’s claim was made over 50 years ago and, as noted by Reviewer 1, Dr. Koonin, most biologists now make at least some use of both evolutionary and functional methods and modes of thought. Even Mayr acknowledged that the two purported aspects of biology “have many points of contact and overlap,” which the authors do acknowledge on p. 5. For example, investigators who would likely be viewed as functional and reductionist in focus routinely make phylogenetic arguments to guide inferences about functional elements of genes and proteins. Finally it is worth noting that the distinction was made primarily to address the nature of causation in biology.

Authors’ response: *We have also used the distinction between functional and evolutionary biology in descriptive terms, or, more precisely, in historical terms. For a certain time lapse, that distinction has had some importance among life scientists.*

**Reviewer’s comment 4.** p. 5 – The sudden introduction of orphan metabolic activities and genes in the context of the closing gap between functional and evolutionary biology seems forced. After this issue, supposedly central for this article, is introduced, it does not reappear for 12 pages. Intervening is a historical discussion that contains little new information and all of the key issues addressed are more thoroughly covered elsewhere.

Authors’ response: *Orphan metabolic activities and genes are not exactly central to this article, whose scope is represented in its title.*

**Reviewer’s comment 5.** pp. 9–12 – I see little value in discussions of one to a few paragraphs of topics such as the supposed distinction between adaptation and exaptation (1 paragraph), niche construction (1 paragraph), the role of cooperation in evolution (1 paragraph), epigenetics (2 paragraphs), and evolutionary developmental biology (2 paragraphs).

Authors’ response: *As specified in the first answer, the concept of exaptation (but also the other aspect considered), developed in the context of evolutionary biology, help functional biologists to properly address observations of multi-functional activities performed by certain proteins. Unprepared minds could regard these unexpected findings as artifacts, instead of possible meaningful discoveries.*

**Reviewer’s comment 6.** pp. 17–18 – The discussions of orphan metabolic activities and genes remain superficial, effectively two paragraphs total, despite the authors statement that they represent a major challenge to evolutionary biology. What could have been useful is a thorough discussion of these challenges and how to address them.

Authors’ response: *Reviews have a limit of 3000 words.*
